# Supplementary material for: Prevalence of child malnutrition and household socioeconomic deprivation: A case study of marginalized district in Punjab, Pakistan
Source: PLoS One. 2022 Mar 10;17(3):e0263470. doi: 10.1371/journal.pone.0263470 (PMC8912173; doi:10.1371/journal.pone.0263470)
Supplement: S1 Table — (PDF) [file pone.0263470.s001.pdf]

Table S1. Distribution of sample size from tehsils to union councils.

District	Tehsil	Union council No	Population of UCs	Sample for UC= Population of 1st UC / total population of 3 UC's* sample of tehsil
Rahimyar	Khanpur	N1= No. of respondent's in UC1	24349	24349/90811*96= 25.47= ~26
Khan		N2= No. of respondent's in UC2	32876	32876/90811*96= 34.39= ~34
		N3= No. of respondent's in UC3	33586	33586/90811*96= 35.14= ~36
		Total 3 UCs population	90811	Household Sample in Tehsil= 96
	Liaquatpur	N4= No. of respondent's in UC4	39367	39367/126573*81= 25.02= ~ 25
		N5= No. of respondent's in UC5	40990	40990/126573*81= 26.23= ~26
		N6= No. of respondent's in UC6	46216	46216/126573*81= 29.57= ~30
		Total 3 UCs population	126573	Household Sample in Tehsil= 81
	Rahimyar Khan	N7= No. of respondent's in UC6	32870	32870/111793*115= 34.34= ~34
		N8= No. of respondent's in UC6	45423	45423/111793*115= 45.51= ~46
		N9= No. of respondent's in UC6	33500	33500/111793*115= 35.15= ~35
		Total 3 UCs population	111793	Household Sample in Tehsil= 115
	Sadiqabad	N10= No. of respondent's in UC6	31543	31543/88105*92= 32.65= ~33
		N11= No. of respondent's in UC6	31269	31269/88105*92= 32.36= ~32
		N12= No. of respondent's in UC6	25293	25293/88105*92= 26.98= ~27
		Total 3 UCs population	88105	Household Sample in Tehsil= 92
Total	4	12		N=384
