# Supplementary material for: Prevalence of child malnutrition and household socioeconomic deprivation: A case study of marginalized district in Punjab, Pakistan
Source: PLoS One. 2022 Mar 10;17(3):e0263470. doi: 10.1371/journal.pone.0263470 (PMC8912173; doi:10.1371/journal.pone.0263470)
Supplement: S2 Table — PDHS- 2017–18 (used for national rates), MICS- 2017–18 (used for provincial rates), and Punjab Development Statistics- 2015 (used for district rates). (PDF) [file pone.0263470.s002.pdf]

Table S2. Comparison of stunting, wasting, underweight prevalence, under-five and infant mortality rates at national, provincial and district levels.

	Stunting	Underweight	Wasting	Infant Mortality Rates per 1000	Under-five Mortality Rate per 1000
Pakistan	38%	23%	8%	62	74
Punjab	31.5%	21.2%	7.5%	60	69
District Rahimyar Khan	46%	42%	19%	98	127

PDHS- 2017-18 (used for national rates), MICS- 2017-18 (used for provincial rates), and Punjab Development Statistics- 2015 (used for district rates).
