# Supplementary material for: Prevalence of child malnutrition and household socioeconomic deprivation: A case study of marginalized district in Punjab, Pakistan
Source: PLoS One. 2022 Mar 10;17(3):e0263470. doi: 10.1371/journal.pone.0263470 (PMC8912173; doi:10.1371/journal.pone.0263470)
Supplement: Attachment — Submitted filename: PLOSE ONE - Editoral Comments.docx [file pone.0263470.s004.docx]

PONE-D-21-20140
Prevalence of Child Malnutrition and Household Socio-Economic Deprivation: An Empirical Analysis in Southern Punjab, Pakistan
Dr Waqar Ameer

Dear Dr Ameer,

Thank you for submitting your manuscript to PLOS ONE. Your manuscript files have been checked in-house and before we can proceed, we need you to address the following issues:

1. During the internal assessment of the article we have noted in your ethics statement : "The present study was approved by the Faculty of Department of Health Economics, Pakistan
290 Institute of Development Economics Islamabad, Pakistan"

Based on this information it is not clear whether an appropriate IRB committee has provided ethics approval. Please ensure that you have clearly indicated the full name of the ethics committee/institutional review board(s) that approved your specific study.

Pakistan Institute of Development Economics (PIDE) Islamabad is a think-tank organization attached with the Ministry of Planning, Development, and Special Initiatives. Its core objective is to do research and provide basis for policy making in the economic development of Pakistan.

To organize and manage a field survey for research, a graduate research management council (GRMC) at PIDE approves the survey protocols. The GMRC at PIDE works as an institutional review board (IRB) in any other research organization. 6th GRMC of PIDE approved study while department of health economics and further department of health District Rahimyar Khan approved survey protocols and tools. In tools we used MUAC tape, weight machine and height measurements tape for collecting data on height, weight, and age of children and mothers.

Furthermore, please attach a copy of the original ethics approval documents as well as an English translate copy as supporting file. Finally, please provide additional details regarding participant consent. In the ethics statement in the Methods and online submission information, please ensure that you have specified whether consent was written or verbal/oral. If consent was verbal/oral, please specify: We had taken verbal consent from the mothers of children before starting the survey.

The letter is attached herewith for your reference and manuscript track changes revision document is also attached in our resubmission to check carefully our grammatical and editing changes to our manuscript.

i) whether the ethics committee approved the verbal/oral consent procedure,

GRMC approved to conduct the survey. It was the principal investigator’s discretion to get written or verbal consent from the respondents before starting interview for data collection. However, principal investigator opt verbal consent.

ii) why written consent could not be obtained, and

Our study examines the child nutrition at rural areas of Southern Punjab. In this geographical zone, overall literacy rate is not satisfactory. In the specific rural locations from where the data was collected, the 74% of the respondent mothers had no formal education. We have also indicated level of education of mothers in the article. Due to lack of formal education, principal investigator decided to go for verbal consent. Secondly, mothers were reluctant in signing or put thumb impression on paper because of lack of knowledge.

iii) how verbal/oral consent was recorded. If your study included minors, please state whether you obtained consent from parents or guardians in these cases. If the need for consent was waived by the ethics committee, please include this information.

At a first stage, after greeting, the lady health workers (enumerator) explained the objective of the survey to the respondent mothers in one of the Saraiki or Punjabi languages. After clarifying the queries, the enumerator invited them to participate by replying to the survey questions. The survey was started if respondent mother verbally agreed to participate.

2. Our editorial staff has assessed your submission, and we have significant concerns about the grammar, usage, and overall readability of the manuscript. We believe that the manuscript is much more likely to be accepted if it is easy for the reviewers to read and understand. We therefore request that you revise the text to fix the grammatical errors and improve the overall readability of the text before we send it for review.

We have improved the text in the article. It is much easier now for the reader to understand.

We suggest you have a fluent, preferably native, English-language speaker thoroughly copyedit your manuscript for language usage, spelling, and grammar. If you do not know anyone who can do this, you may wish to consider employing a professional scientific editing service.

Language and editing was done by Dr. Waqar Ameer and Dr. Waqar Ameer is fluent and quite good English-language speaker.

Whilst you may use any professional scientific editing service of your choice, PLOS has partnered with both American Journal Experts (AJE) and Editage to provide discounted services to PLOS authors. Both organizations have experience helping authors meet PLOS guidelines and can provide language editing, translation, manuscript formatting, and figure formatting to ensure your manuscript meets our submission guidelines. To take advantage of our partnership with AJE, visit the AJE website (<https://www.aje.com/go/plos/>) for a 15% discount off AJE services. To take advantage of our partnership with Editage, visit the Editage website ([www.editage.com](http://www.editage.com/)) and enter referral code PLOSEDIT for a 15% discount off Editage services. If the PLOS editorial team finds any language issues in text that either AJE or Editage has edited, the service provider will re-edit the text for free.

Please note that having the manuscript copyedited by any editing service does not guarantee selection for peer review or acceptance for publication

Upon resubmission, please provide the following:

- The name of the colleague or the details of the professional service that edited your manuscript

Dr. Waqar Ameer (PhD)

Associate Professor,

School of Economics,

Shandong Technology and Business University, P.R.China

Post Doctoral Research Fellow, School of Economics,

Hunan University, P.R.China

MSc School of Economics,

University of Sheffield, UK

- A copy of your manuscript showing your changes by either highlighting them or using track changes (uploaded as a **supporting information** file)
- A clean copy of the edited manuscript (uploaded as the new **manuscript** file)
- If the language is not sufficiently improved, we may have no choice but to reject the manuscript without review.

We ask that you address this request within 21 days. If you require additional time, please email the journal office. We are happy to grant extensions of up to one month past this due date. If we have not heard from you within 21 days, your manuscript will be withdrawn from Editorial Manager.

Thank you for considering PLOS ONE for publication of your manuscript.

Sincerely,

Liz McLaren
PLOS ONE
